# Prenatal Exposure to Arsenic and Cadmium Impacts Infectious Disease-Related Genes within the Glucocorticoid Receptor Signal Transduction Pathway

**DOI:** 10.3390/ijms151222374

**Published:** 2014-12-03

**Authors:** Julia E. Rager, Andrew Yosim, Rebecca C. Fry

**Affiliations:** 1 Department of Environmental Sciences and Engineering, Gillings School of Global Public Health, University of North Carolina, 135 Dauer Drive, CB 7431, Chapel Hill, NC 27599, USA; E-Mails: jrager@live.unc.edu (J.E.R.); yosim@live.unc.edu (A.Y.); 2 Curriculum in Toxicology, School of Medicine, University of North Carolina, 104 Mason Farm Road, CB 7310, Chapel Hill, NC 27599, USA

**Keywords:** arsenic, cadmium, environmental toxicant, epigenome, genome, glucocorticoid receptor, infectious disease, *in utero*, pathway, signal transduction

## Abstract

There is increasing evidence that environmental agents mediate susceptibility to infectious disease. Studies support the impact of prenatal/early life exposure to the environmental metals inorganic arsenic (iAs) and cadmium (Cd) on increased risk for susceptibility to infection. The specific biological mechanisms that underlie such exposure-mediated effects remain understudied. This research aimed to identify key genes/signal transduction pathways that associate prenatal exposure to these toxic metals with changes in infectious disease susceptibility using a Comparative Genomic Enrichment Method (CGEM). Using CGEM an infectious disease gene (IDG) database was developed comprising 1085 genes with known roles in viral, bacterial, and parasitic disease pathways. Subsequently, datasets collected from human pregnancy cohorts exposed to iAs or Cd were examined in relationship to the IDGs, specifically focusing on data representing epigenetic modifications (5-methyl cytosine), genomic perturbations (mRNA expression), and proteomic shifts (protein expression). A set of 82 infection and exposure-related genes was identified and found to be enriched for their role in the glucocorticoid receptor signal transduction pathway. Given their common identification across numerous human cohorts and their known toxicological role in disease, the identified genes within the glucocorticoid signal transduction pathway may underlie altered infectious disease susceptibility associated with prenatal exposures to the toxic metals iAs and Cd in humans.

## 1. Introduction

According to the World Health Organization, infectious diseases are primary contributors to the global burden of disease currently estimated to result in hundreds of millions of disability-adjusted life years worldwide [1,2]. Infectious diseases are amongst the leading causes of mortality, responsible for approximately 16% of total global deaths each year [3]. While much research has been carried out to understand the relationship between infectious disease patterns and human behavior [4,5], the role that environmental contaminants play as agents that alter infectious disease susceptibility is under-recognized.

There is increasing evidence that exposure to environmental contaminants influences or disrupts the host defense responses to infectious agents, namely the innate and adaptive immune systems and their associated inflammatory response pathways. Alterations in the expression or activity of proteins within the immune system can influence susceptibility to infection from viral, bacterial, or parasitic diseases. As specific environmental examples, cigarette smoke [6], mercury [7], and organophosphates [8] are established immunomodulators that can increase susceptibility to infectious diseases under conditions of chronic exposure in adults.

In addition to the deleterious health outcomes associated with chronic exposure of adults to environmental contaminants, *in utero* exposures can harm the developing fetus disrupting host defenses and altering appropriate responses to infectious agents. There is mounting evidence related to developmental toxicity of *in utero* exposures to the world-wide poisons inorganic arsenic (iAs) and cadmium (Cd), currently ranked amongst the highest prioritized hazardous substances in the U.S. [9]. Current estimates suggest that more than 100 million individuals worldwide are exposed to iAs at levels associated with adverse health outcomes [10]. This is of concern as chronic exposure to iAs, as well as Cd, has been associated with both cancer and non-cancer endpoints in adults [11,12]. Additionally, *in utero* and early life exposure to iAs can cause detrimental impacts on fetal and childhood development and increase the risk for certain diseases/disorders later in life, including respiratory problems, cardiovascular disease, and cancer [13,14,15,16,17,18]. The evidence supporting these relationships was largely informed by population studies of prenatal iAs exposure in Antofagasta, Chile. Specifically, increased incidence of morbidity and mortality from cancer and non-cancer endpoints were observed up to forty years after prenatal/early childhood exposures to iAs [13,18,19,20]. These data highlight the long-lasting health consequences of this early life exposure to iAs. In addition to the later life health consequences of exposure, prenatal exposure to both iAs or Cd have been associated with detrimental health effects at birth including risk of low birth weight [21,22,23]. Being born at lower birth weight puts infants at subsequent increased risk for diseases including those that are associated with infectious agents [24].

Directly relevant to the research presented here, prenatal iAs exposure has also been associated with increased susceptibility to infectious diseases in infants. Several studies of Bangladeshi infants have demonstrated that elevated levels of prenatal iAs increases the risk of diarrhea and upper and lower respiratory infections [25,26]. Such findings have also been replicated in areas where iAs levels are lower than in Bangladesh. For instance, infants in New Hampshire, U.S., who experienced elevated exposure to iAs *in utero* displayed increased severity of respiratory tract infections [27]. While a specific mechanism for the prenatal iAs-associated increased susceptibility to infection is not established, it has been shown that such exposure reduces fetal and childhood thymic function through a reduction in naive T cells [28] potentially resulting in immunodeficiency. While studies have yet to investigate the influence of prenatal iAs exposure on infectious disease susceptibility later in adulthood, it is likely that iAs-associated increased risk for infection extends beyond infancy. While human studies on risk of infection associated with prenatal Cd have not been carried out, rodent studies have supported that prenatal Cd exposure alters the immune system [29,30].

To begin to fill the knowledge gap of specific genes/pathways that underlie prenatal iAs or Cd exposure-induced susceptibility to infectious disease, we developed and employed the Comparative Genomic Enrichment Method (CGEM). Using this approach, a set of exposure and infectious disease-related genes that were enriched for pathways of high priority were identified. Given their toxicological role in responses to infectious agents and their modulation across numerous studies, these pathways are likely important contributors to environmental exposure-induced immunomodulation underlying response to infectious agents.

## 2. Results

### 2.1. The Comparative Genomic Enrichment Method (CGEM)

**Figure 1:**
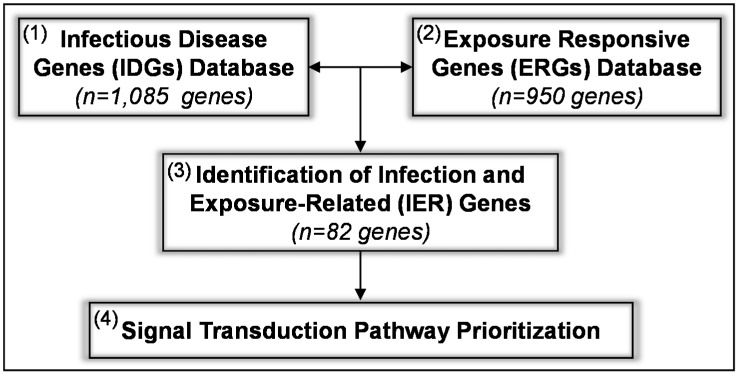
The Comparative Genomic Enrichment Method (CGEM) was used to identify key genes and proteins and their corresponding signal transduction pathways likely involved in exposure-associated modifications in infectious disease-related signaling. The CGEM consists of four steps: (1) Identification of the infectious disease gene (IDG) database; (2) Identification of the exposure responsive gene (ERG) database; (3) Comparison of the IDG and ERG databases to identify infection and exposure-related (IER) genes; and (4) Systems-level processing to prioritize pathways enriched within the IER gene database.

The CGEM is a four-tiered approach (Figure 1) comprising the following steps: (1) Generation of a database consisting of genes/proteins with known roles in infectious disease signaling termed infectious disease genes (IDGs); (2) Development of a database of genes/proteins that are modified at an epigenomic, genomic, or proteomic level in response to prenatal iAs or Cd exposure in human pregnancy cohorts termed exposure responsive genes (ERGs); (3) An analytical comparison of the IDG and ERG databases resulting in the identification of a database of infection and exposure-related (IER) genes; and (4) Systems-level analysis to identify pathways prioritized for their likely involvement in infection and exposure-related responses.

The CGEM was used in the present study to elucidate key biological pathways likely involved in environmental exposure-associated changes in infectious disease-related signaling, using prenatal exposure to arsenic and cadmium as the exposures of interest.

### 2.2. Application of the CGEM Identifies Infection and Exposure-Related (IER) Genes

In order to evaluate whether there are a common set of genes/proteins related to infectious disease that also overlap with genes that are associated with prenatal exposures to iAs or Cd, a database of *n* = 1085 unique infectious disease genes (IDGs) was developed (Table S1). Specifically the IDG database was developed by integrating genes/proteins representing 23 pathways related to viral, bacterial, and parasitic infection. To note, amongst these 1085 genes are a set of 15 genes with single nucleotide polymorphisms (SNPs) known to influence response to infectious disease [31] (Table S1).

A separate exposure responsive gene (ERG) database was developed consisting of *n* = 950 unique genes obtained through a cross-study analysis (Table S2). Specifically, these genes have been identified to have altered levels of 5-methyl cytosine methylation, altered mRNA abundance and/or altered protein expression levels associated with prenatal exposure to iAs and/or Cd in twelve human pregnancy cohorts. To note, environmental exposures can cause shifts in immune cell populations [32], and some of the human pregnancy cohort studies did not report to have controlled for the impact of white blood cell shifts in response to iAs or Cd. Still, many of these prior studies did control for the impact of white blood cell shifts [33,34,35,36]. For instance, Koestler* et al.* used a statistical methodology, referred to as a cell mixture deconvolution methodology, to infer changes in cord blood leukocyte distributions between exposure quartiles using DNA methylation signatures [34]. In addition, Rager* et al.*, Rojas* et al.*, and Sanders *et al*. compared genes identified as iAs/Cd-associated to previously published lists of immune cell subset-specific genes [33,35,36]. Rager* et al.* found a small percentage (2%) of iAs-associated genes with expression levels specific to immune cell subsets [36], while Rojas* et al.* did not find any iAs-associated genes with differential methylation specific to immune cell subsets [35]. In the study by Sanders* et al.*, only one gene with differential methylation associated with Cd was specific to an immune cell subtype [33]. Thus the observed changes in DNA methylation, mRNA abundance, and/or protein expression are not reflective of changes in immune cell populations.

The metalloid/metal iAs and Cd were selected for evaluation, as there is substantial literature relating prenatal exposure in human cohort studies with changes to the epigenome, genome, and/or proteome. It is important to consider for these studies that while iAs tends to more readily cross the placental barrier, cord blood concentrations of both iAs and Cd increase as maternal exposure increases [21,37,38], thus fetal exposure during critical times of development is occurring. The studies used to generate the ERG database assessed human populations with iAs exposure levels comparable to those that have been associated with increased risk of childhood infection (Table 1 and Table 2). To our knowledge, this represents all studies to date where gene-specific information could be compiled from pregnancy cohorts and where data overlapped with the IDGs.

**Table 1:** iAs and Cd levels measured in human pregnancy cohort studies.

Study	Metalloid/Metal	Biological Media	Measurements in Biological Media ^a^	Measurements in Drinking Water ^a^
[39]	iAs	Urine ^b^	GW8: 136 µg/L (26–341) ^c,d^	Not Reported
			GW30: 143 µg/L (27–334) ^c,d^	
[16]	iAs	Urine ^b^	64.5 µg/L (6.2–319.7) ^e^	51.7 µg/L (ND–326)
[40]	iAs	Urine ^b^	GW5–14: Median: 66 µg/L (3–740) ^d^	Not Reported
			GW26–36: Median: 89 µg/L	
[41]	iAs	Urine ^f^	GW24–28: Median: 4.4 μg/L (1.8–11.9) ^e^	0.36 µg/L (0.02–3.55) ^g^
[42]	iAs	Toenail Clippings	4.8 µg/g (0.1–68.63)	Not Reported
[43]	iAs	Cord blood	5.79 µg/g (1.31–10.37) ^d,e^	8.38 µg/L (0.17–61.63)
		Toenail Clippings	1.52 µg/g (ND–8.23) ^e^	
		Fingernail Clippings	1.91 µg/g (ND–9.08) ^d,e^	
		Hair Clippings	0.05 µg/g (ND–0.38) ^e^	
[44]	iAs	Urine ^b^	GW ≤ 28: 12.35 µg/L (0.05–260.3)	14.8 µg/L (1–230)
[34]	iAs	Urine ^b^	GW24–28: Median: 4.1 µg/L (0.45–300)	1.2 µg/L (0.03–100)
[36]	iAs	Urine ^b^	64.5 µg/L (6.2–319.7) ^e^	51.7 µg/L (ND–326)
[35]	iAs	Urine ^b^	64.5 µg/L (6.2–319.7) ^e^	51.7 µg/L (ND–326)
[33]	Cd	Peripheral Blood	0.44 µg/L (ND–1.05) ^e^	Not Reported
[45]	Cd	Urine	Median Urine GW8: 0.77 µg/L (0.25–2.4) ^h^	Not Reported
		Peripheral Blood	Median Blood GW14: 1.3 (0.54–3.1) ^d,h^ µg/kg	

^a^ Mean (range), unless otherwise noted; ^b^ Measurement of total urinary arsenic (U-tAs). U-tAs was defined as the sum of iAs (As^III^ + As^V^), DMA^III^, DMA^V^, MMA^III^, and MMA^V^; ^c^ 1st–4th Quartile range; ^d^ Measurement utilized for statistical analysis results; ^e^ Collected at time of delivery; ^f^ Measurement of total urinary arsenic (U-tAs). U-tAs was defined as the sum of iAs (As^III^ + As^V^), DMA^V^, and MMA^V^; ^g^ Inter-quartile range; ^h^ 5th–95th Percentile range; ND, Non-detectable; and GW, Gestational week.

**Table 2:** iAs levels and health endpoints evaluated in human cohorts providing evidence for increased risk of infectious disease.

Study	Metalloid	Biological Media	Measurements in Biological Media ^a^	Measurements in Drinking Water ^a^	Health Endpoint	Duration of Health Endpoint Observation
[25]	iAs	Urine ^b^	GW8: 152 µg/L (1–1211)	Not Reported	LRTI, Severe	Mean: 75 Days
			GW30: 166 µg/L (2–1440)		LRTI, Diarrhea	
[26]	iAs	Urine ^b^	GW6–10: 152 µg/L (1–2020)	Not Reported	ARI ^c^	12 Months
			GW30: 146 µg/L (4–1126)			
[15]	iAs	Urine ^d^	GW24–28: 6 µg/L (0.45–58.3)	5.2 µg/L (0.01–67.5)	Infection ^e^, LRTI, URTI	4 Months

^a^ Mean (range); ^b^ Measurement of total urinary arsenic (U-tAs). U-tAs was defined as the sum of iAs (As^III^ + As^V^), DMA^III^, DMA^V^, MMA^III^, and MMA^V^; ^c^ ARI only statistically significant in males; ^d^ Measurement of total urinary arsenic (U-tAs). U-tAs was defined as the sum of iAs (As^III^ + As^V^), DMA^V^, and MMA^V^; ^e^ Infection requiring a doctor visit or treatment with prescription medication; ARI, Acute respiratory infection; GW, Gestational week; LRTI, Lower respiratory tract infection; and URTI, Upper respiratory tract infection.

Comparing the list of IDGs against the ERGs identified a common set of 82 genes representing those that play a role both in infectious disease-related signaling as well as those that are targets for modulation/modification under conditions of prenatal iAs or Cd exposure, hitherto referred to as infection and exposure-related (IER) genes (Table S3). Additionally, 16 of the 82 (20%) IER genes are known to play a role in all three major infectious disease pathways, viral, bacterial, and parasitic pathways, including interferon γ receptor 1 (*IFNGR1*), interferon γ (*IFNG*), tumor necrosis factor (*TNF*), caspase 9, apoptosis-related cysteine peptidase (*CASP9*), FBJ murine osteosarcoma viral oncogene homolog (*FOS*), major histocompatibility complex, class II, DP α 1 (*HLA-DPA1*), major histocompatibility complex, class II, DQ α 1 (*HLA-DQA1*), intercellular adhesion molecule 1 (*ICAM1*), interleukin 1 β (*IL-1β*), interleukin 8 (*IL8*), jun proto-oncogene (*JUN*), nuclear factor of κ light polypeptide gene enhancer in B-cells 1 (*NF-κB1*), nuclear factor of κ light polypeptide gene enhancer in B-cells inhibitor, α (*NF-κBIA*), phosphoinositide-3-kinase, regulatory subunit 1 (α) (*PIK3R1*), signal transducer and activator of transcription 1 (*STAT1*), and toll-like receptor 9 (*TLR9*) (Table S3).

### 2.3. The IER Genes Are Enriched for Involvement in the Glucocorticoid Receptor (GR) Signal Transduction Pathway

**Figure 2:**
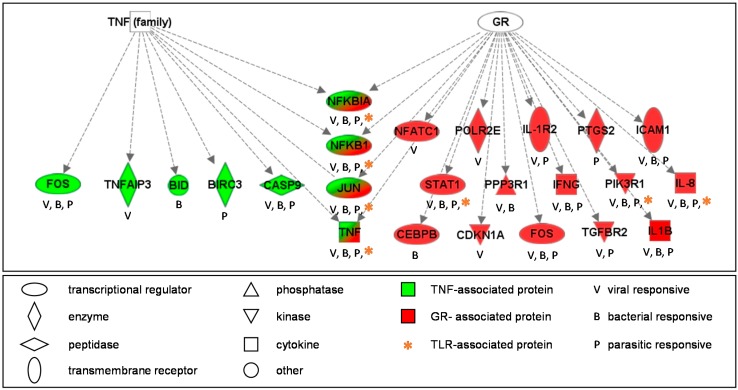
The glucocorticoid receptor (GR) signal transduction pathway is enriched within the infection and exposure-related gene networks. Proteins encoded by the IER genes are shown for those directly involved in the GR pathway and the associated tumor necrosis factor (TNF) pathway.

In order to determine whether the IER genes enrich for specific biological pathways, a systems-level analysis was carried out using the 82 IER genes. Perhaps not surprisingly, given the IDG database into which the information was compiled, the analysis identified enrichment for canonical pathways highly relevant to infectious disease and inflammatory response signaling. Interestingly, many of the IER genes were identified to belong to the glucocorticoid receptor (GR) signal transduction pathway (*p* = 1.58 × 10^−18^) where 19 of the 82 IER genes are known to be involved. Another GR-associated pathway was also enriched, namely the tumor necrosis factor receptor 1 (TNFR1) (*p* = 5.01 × 10^−13^) pathway, where nine of the 82 IER genes were present. Also relevant to the GR signal transduction pathway, the toll-like receptor (TLR) pathway was enriched (*p* = 6.31 × 10^−13^) wherein eight of the IER genes were present (Figure 2, Table S4).

The IER genes were analyzed for their known associations with potential cellular regulators including, but not limited to, transcription factors, signaling molecules, and drugs. The 82 IER genes were enriched for their association with numerous upstream regulators including the following six most significant regulators: Lipopolysaccharide (LPS) (*p* = 4.98 × 10^−38^), colony stimulating factor 2 (CSF2) (*p* = 2.23 × 10^−30^), TNF (*p* = 9.72 × 10^−30^), polyinosinic-polycytidylic acid (poly(I:C)) (*p* = 1.18 × 10^−28^), IL1β (*p* = 4.74 × 10^−27^), and NF-κB (*p* = 1.56 × 10^−24^). Of note, LPS has been shown to be associated with 49 of the 82 (60%) IER genes (Table S5). Further supporting both the pathway and predicted upstream regulators analysis, a separate analysis of the DNA sequences within the promoter regions of the IER genes also identified an enrichment for genes with binding sites for the GR, specifically containing “negative” glucocoticoid response elements (NGRE) (3.91 × 10^−6^), as well as sites bound by the NF-κB transcription factor family (3.72 × 10^−14^) (Table S6).

### 2.4. Validation of ERG-Association with iAs and Cd Using the Comparative Toxicogenomics Database (CTD)

As validation of the relationship between the ERGs and iAs and Cd, the CTD was queried. This manually-curated database is specific for environmental contaminants and their relationships to genes including perturbations at the mRNA and protein level collected from published toxicological and epidemiological studies. At the time of our analyses, the CTD analyzed over 95,000 studies to derive over 15 million toxicogenomic relationships between approximately 11,000 chemicals, 27,000 genes, and 5900 diseases [46]. Thus the CTD includes data from both* in vitro* and* in vivo* animal model-based systems while the ERG list was compiled solely from data derived from human pregnancy cohorts.

Supporting the analytical approach, the CTD contained information that 50 of the 82 (61%) IER genes have been shown to be modulated by iAs/iAs metabolites and 34 of the 82 (41%) IER genes have been shown to be modulated by Cd in other studies (Table S7). These percentages of genes are much higher than those produced using a randomly generated gene set of the same size (data not shown). It is important to note as further validation of the relationship between iAs and Cd and the IER genes that many of the studies within CTD evaluated the effects of exposure using cells/animals that were treated with iAs/Cd and compared their responses to untreated, negative controls. For instance, an* in vitro* study within CTD using human bronchial epithelial cells compared gene expression profiles from unexposed cells* versus* cells exposed to iAs and iAs metabolites and identified four genes associated with exposure that are contained in our IER gene list: BH3 interacting domain death agonist (*BID*), *NF-κB1*, *PIK3R1*, and tumor protein p53 (*TP53*) [47]. Additionally, many of the IER genes have been shown to be modulated in response to exposure to a range of other environmental toxicants including toxic metals such as beryllium, chromium, cobalt, copper, lead, mercury, nickel, selenium, and zinc. Interestingly there is also evidence for association of the IER genes with other non-metal toxicants including acrolein, asbestos, atrazine, benzene, benzo(a)pyrene, bisphenol A, estradiol, formaldehyde, nitric oxides, particulate matter, and silicon dioxide (Table S7). These data support that, in addition to iAs and Cd, the identified set of 82 IER genes are dysregulated by other environmental contaminants in various model systems. These findings have implications for the putative role that these contaminants play in environmentally-induced infectious disease susceptibility.

## 3. Discussion

Current estimates suggest that millions of pregnant women are exposed to harmful levels of toxic metalloids/metals such as iAs and Cd putting their developing fetuses at risk [10,11,12,21]. Such exposures are associated not only with detrimental health effects observable at birth, but also with increased risk for infant susceptibility to diseases caused by infectious agents. As a result, in areas with high iAs/Cd exposure, endemic patterns of infectious disease are likely greatly influenced by the presence and exposure to these environmental contaminants. Nevertheless, specific genes/pathways altered in their cellular signaling capacities that may underlie these associations are currently unknown, thus hindering opportunities for prevention or clinical treatment of disease. To prioritize signal transduction pathways that likely mediate these relationships, the CGEM was developed to integrate information from an infectious disease database and an exposure responsive database compiled from a compendium of pregnancy cohort studies. For analysis, the pregnancy cohort studies were required to have assessed gene-specific information on iAs or Cd-associated changes in DNA methylation levels, mRNA expression levels, and/or protein levels. This CGEM approach resulted in the identification of 82 genes associated with prenatal iAs/Cd exposure and infectious disease responses. Amongst these genes were 19 genes that are involved in the GR signal transduction pathway (*p* = 1.58 × 10^−18^) as well as enriched for the presence of specific binding elements for the GR transcription factor. These data support the novel finding that the GR signal transduction pathway is a likely contributor to prenatal iAs/Cd-associated infectious disease susceptibility.

The GR signal transduction pathway is known to respond to glucocorticoids released by the hypothalamic-pituitary-adrenal (HPA) axis [48]. Transcription of target genes is modulated through direct binding of DNA sequences at glucocorticoid response elements (GRE) or negative glucocorticoid response elements (nGRE). Exogenous chemicals that disrupt the GR signal transduction pathway and alter homeostatic gene expression patterns may influence disease susceptibility through disruptions in immune-related pathways [49]. As evidence for this, disruptions to the HPA axis or glucocorticoid levels increase rates of infection and toxicity in animal models [48]. In the context of the environmental contaminants of interest here, both iAs and Cd have been previously shown to influence the GR [50,51], where iAs in particular has been shown to alter GR function and GR-dependent gene expression [51,52,53]. In support of the role that the GR pathway plays in mediating toxicity to iAs and Cd, we have recently demonstrated that chemical inhibition of GR signaling protects against iAs and Cd-induced cellular toxicity* in vitro*, and that GR inhibition protects against iAs-induced teratogenesis [51]. With the findings from the present study, we hypothesize that the GR pathway not only influences metal-induced birth defects, but may mediate the effects of iAs and Cd on infectious disease susceptibility. Future studies will test this hypothesis directly and evaluate the impact of GR signaling as a mediator of metals-associated changes in immune function.

Amongst the identified proteins that are known to interact with the GR pathway was IFNG [54,55], a member of the 16 IDGs known to play a role in responding to viral, bacterial, and parasitic exposures. Prenatal iAs exposure has been associated with altered IFNG cytokine levels [39] as well as *IFNGR1* gene expression [42], thus represented in two of the twelve studies of interest here. The ability of iAs and potentially other environmental contaminants to influence *IFNG* expression is of concern as it is a critical regulator of a host’s immune and inflammatory response [56]. IFNG plays a role in infectious disease pathogenesis where polymorphisms have been shown to increase risk for infectious diseases including tuberculosis [57], malaria [58], and Chagas disease [59]. These polymorphisms are often correlated with varying *IFNG* expression levels, where increased or decreased expression levels have been tied both to infectious disease risk and severity. Given the ties between iAs/Cd exposure, IFNG, and infectious disease, it is plausible that it represents a key player related to the GR pathway that underlies iAs/Cd exposure and disease susceptibility.

The GR signal transduction pathway is highly integrated in the cell, known to influence transcription indirectly through interactions with NF-κB, TLR, and TNF [48,60,61], all of which were represented in the IER gene set. TNF signaling is a critical player in inflammatory response signaling and immune function [62] with increased expression following exposure to lipopolysaccharides (LPS) [63]. Interestingly, 49 of the 82 (60%) IER genes have known associations with LPS. In relationship to disease, TNF polymorphisms are associated with susceptibility to a range of infectious diseases including mucocutaneous leishmaniasis [64], *Chlamydia trachomatis* [65], cerebral malaria [66], and lepromatous leprosy [67]. Polymorphisms in *TNF* can impact its expression directly altering the immune system impacting disease severity/susceptibility [68]. Of relevance to the environmental metals/metalloid under study here, TNF polymorphisms have also been associated with iAs-associated skin lesions and respiratory disease [69]. Similarly, altered TNF transcript and protein expression has been related to Cd-induced liver toxicity [70]. Taken together, an important consideration is that environmental contaminants that impact TNF expression may influence infectious disease susceptibility. Thus, TNF represents an additional GR-related protein and gene target for future mechanistic investigations relating environmental contaminant and infectious disease relationships.

While supporting the novel finding that the GR pathway may be a key component underlying metals-induced immune dysfunction, this study is not without limitations. The endpoints that were selected for evaluation here (*i.e.*, DNA methylation, mRNA abundance, and protein expression) can be transient in their response to toxic agents. Importantly, however, there is evidence that some of the mRNA expression changes associated with toxicant exposure have been identified as reversible but others were irreversible [71]. As further support for permanent changes induced by metals during the *in utero* period, adult mice that were exposed to iAs during gestation have shown sustained mRNA expression changes later in life related to carcinogenesis in the liver [72]. DNA methylation changes associated with environmental stressors can also be permanent, persisting through multiple generations [73]. Future research will establish whether the genes that are represented within the GR pathway are stably modified under conditions of *in utero* exposure.

There is evidence from this research that many of the iAs and Cd-associated genes are not solely responsive to these exposures as they are also perturbed by other metals present in the environment such as beryllium, chromium, cobalt, copper, lead, mercury, nickel, selenium, and zinc. Indeed, previous studies have reported that multiple metals, including iAs, beryllium, Cd, chromium, lead, mercury, selenium, and zinc, influence signaling of the GR pathway [51,74]. Additionally the IER genes are also known to be impacted by a range of other environmental contaminants such as asbestos, benzene, benzo(a)pyrene, bisphenol A, estradiol, formaldehyde, nitric oxides, particulate matter, and silicon dioxide. Thus, the data highlight that in addition to prenatal iAs and Cd exposure in humans, many other environmental contaminants indeed impact signaling of the infectious disease-related genes. As data on prenatal exposure to these contaminants in human populations are currently limited, future studies will use CGEM to assess the relationship between prenatal exposure and IER genes pending the availability of genomic, epigenomic, and proteomic data from human cohorts. The current study’s findings are important given the extent of worldwide exposure to toxic substances and thus the potential for the GR pathway to mediate responses to infectious agents, representing a clear target for disease intervention.

## 4. Experimental Section

### 4.1. Identifying Infectious Disease Genes (IDGs)

To generate a database of key genes involved in infectious disease-related signaling, infectious disease genes (IDGs) were compiled including genes encoding proteins involved in canonical pathways relevant to infectious disease signaling. A total of 23 Kyoto Encyclopedia of Genes and Genomes (KEGG) [75] viral, bacterial and parasitic infectious diseases pathways were included: Vibrio cholera, *Helicobacter pylori*, pathogenic *Escherichia coli*, *Salmonella*, *Shigellosis*, *pertussis*, legionellosis, *Staphylococcus aureus*, tuberculosis, generalized bacterial invasion of epithelial cells, human T-lymphotropic virus, measles, influenza A, hepatitis B, hepatitis C, herpes simplex, epstein-Barr virus, amoebiasis, malaria, toxoplasmosis, leishmaniasis, African trypanosomiasis, and American trypanosomiasis. These genes were associated with the above mentioned KEGG infectious disease pathways to produce a final list of *n* = 1085 IDGs.

### 4.2. Identifying Exposure Responsive Genes (ERGs)

In order to assess the relationship between iAs or Cd exposure and infectious disease, the database of IDGs was compared with genes previously altered in response to prenatal exposure to iAs or Cd. Specifically, twelve datasets were gathered from previous human cohort studies evaluating mRNA changes, DNA methylation changes, or protein expression changes associated with prenatal exposure to iAs or Cd. Five of the twelve gene lists comprise those with altered gene (mRNA) or those that encoded proteins with expression levels associated with *in utero* exposure to iAs: (1) Fry* et al.*, identified 447 differentially expressed genes from a genome-wide analysis in cord blood leukocytes of 32 pregnant women from the Ron Pibul and Bangkok districts of Thailand [42]; (2) Ahmed* et al.*, performed a gene-specific analysis of 18 inflammation-associated cytokines in cord blood leukocytes and found three differentially expressed proteins in a cohort of 130 pregnant women from Matlab, Bangladesh [39]; (3) Rager* et al.*, conducted a genome-wide analysis in cord blood leukocytes and found 334 differentially expressed mRNAs in a cohort of 40 mother-baby pairs from the Biomarkers of Exposure to ARsenic (BEAR) pregnancy cohort in Gómez Palacio, Mexico [36]; (4) Fei* et al.*, investigated the expression of nine predicted As-associated genes in placental tissue from 133 pregnant women from New Hampshire, U.S [41]; and (5) Bailey* et al.*, investigated As-associated changes to the prenatal proteome and identified 111 proteins associated with prenatal As exposure in the cord blood serum or plasma of 50 newborns from the BEAR cohort in Gómez Palacio, Mexico [76]. Seven of the twelve gene lists comprise genes identified in studies assessing DNA methylation levels: (1) Kippler* et al.*, analyzed genome-wide DNA methylation of cord blood leukocytes in response to Cd exposure from 127 mother-baby pairs from Matlab, Bangladesh and identified 54 genes with differential methylation most associated with Cd [45]; (2) Kile* et al.*, analyzed global DNA methylation and two gene-specific tumor suppressors of cord blood leukocytes in response to prenatal iAs in 113 mother-baby pairs from Sirajdikhan Upazila, Bangladesh [44]; (3) Intarasunanont* et al.*, conducted a global DNA methylation and TP53 specific analysis of cord blood leukocytes from 71 newborns with prenatal iAs exposure from southern Thailand [43]; (4) Koestler* et al.*, performed a genome-wide methylation study of cord blood leukocytes from 134 infants nested in the New Hampshire Birth Cohort Study (NHBCS) and identified 68,353 CpG loci associated with iAs exposure [34]; (5) Sanders* et al.*, performed a genome-wide gene-specific DNA methylation study of 17 mother-baby pairs from Durham, North Carolina and found 61 genes differentially methylated in cord blood leukocytes from prenatal Cd exposure [33]; (6) Broberg* et al.*, carried out a genome-wide methylation analysis of cord blood leukocytes from 127 infants from a pregnancy cohort in Matlab, Bangladesh and identified three CpG sites in boys, but none in girls, that were significantly associated with As exposure after adjustment for multiple comparisons [40]; and (7) Rojas* et al.*, conducted a genome-wide methylation analysis of 38 mother-baby pairs from the previously mentioned BEAR cohort in Gómez Palacio, Mexico and identified 54 genes that were both differentially methylated and differentially expressed in response to iAs in cord blood leukocytes. In total, all twelve gene sets were compiled to form the exposure responsive genes (ERGs) database and compared against the IDGs, where genes in common between the IDG and ERG lists were termed the infection and exposure-related (IER) genes.

Of the twelve studies’ gene sets used to generate the ERG database, nine contained genes within the IDG database and were thus included in the final IER gene database. All nine of these studies evaluated gene expression, DNA methylation, or protein expression within human cord blood leukocytes [33,35,36,39,42,43,44,45,76]. It is notable that some of these published studies included weaknesses in design and implementation. Possible limitations include small sample size and incomplete analysis of possible confounders, such as other environmental exposures. Additionally, variations between studies, including exposure duration and demographic information (e.g., subject age, gender, and race), may influence the reported relationships between prenatal exposure and genomic/epigenomic/proteomic alterations. The iAs and Cd exposure levels found in these studies presented a range of levels, and the studies utilized various biological medias to measure for metals exposure including blood, urine, and nail clippings (Table 1). Still, many of the ERGs (*n* = 67 genes) overlapped across studies, indicating consistency amongst findings and supporting biological plausibility.

### 4.3. Pathway and Upstream Regulator Analysis

Pathway analysis was performed to understand the systems-level relationships amongst the IER genes. For this analysis, IER genes were assessed for their associations to canonical pathways, enabled through Ingenuity Pathway Analysis (IPA) (Ingenuity Systems^®^, Redwood City, CA, USA). Canonical pathways were identified as enriched using the right-tailed Fisher’s Exact test, as performed previously [33], where significance was set at *p* < 0.0001.

An upstream regulator analysis was also carried out using IPA, where transcription factors, growth factors, cytokines, chemicals, drugs, and other molecules with known influences on the IER genes were analyzed as potential regulators. Using a separate *in silico* database that focuses on transcription factors, the IER genes were analyzed for overrepresented transcription factor binding site enrichment using Genomatix’s Overrepresented TFBS tool (Genomatix Software Inc., Ann Arbor, MI, USA) to determine if the IER genes are potentially regulated by common transcription factors [77]. Promoter regions of the IER genes were selected with preference for experimentally verified 5' complete transcripts, number of cap analysis gene expression (CAGE) tags, and relevant transcripts. Promoter regions were defined as 500 base pairs upstream and 100 base pairs downstream of the transcription start site. Resulting transcription factor families that were significantly (*z*-score > 1.96, *p*-value < 0.05) enriched for binding sites within the IER genes were identified.

### 4.4. Validating Gene-Environment Interactions with an Alternative Genomics Database

Gene-chemical interactions were assessed for the IER genes using data retrieved from the Comparative Toxicogenomics Database (CTD) (North Carolina State University, Raleigh, NC, USA) [46]. The CTD curates known interactions between environmental chemicals, genes, and known relationships to disease. As of 2012, the CTD had analyzed over 95,000 studies to derive over 15 million toxicogenomic relationships between approximately 11,000 chemicals, 27,000 genes, and 5900 diseases [46].

## 5. Conclusions

In conclusion, we identified genes that act directly within the GR signal transduction pathway as well as GR-associated genes including *TNF* and *IFNG* that have both been associated with prenatal iAs or Cd exposure in humans and established to play a role in infectious disease. These genes represent novel cellular targets for investigations of gene-environment associations as mediators of contaminant-associated susceptibilities to infectious agents. This study implemented CGEM to extract important information regarding relationships between infectious disease genes and environmental exposures during pregnancy. Such a methodology could not have been carried out until recently, enabled here as a result of the recent advancements in high-throughput platforms applied to human pregnancy cohorts. This approach allows for mechanistic information to be extracted from environment-disease databases to increase the current capacity for the identification of genes that underlie infectious disease susceptibility. The identification of environmental risk factors that contribute to infectious disease susceptibility such as exposure to iAs and Cd, and the knowledge of their targeted genes, would have significant impact on existing strategies for disease prevention.

## Supplementary Materials

Supplementary materials can be found at http://www.mdpi.com/1422-0067/15/12/22374/s1.
